# Altered balance of interleukin-13/interferon-gamma contributes to lacrimal gland destruction and secretory dysfunction in CD25 knockout model of Sjögren’s syndrome

**DOI:** 10.1186/s13075-015-0582-9

**Published:** 2015-03-10

**Authors:** Fang Bian, Flavia L Barbosa, Rosa M Corrales, Flavia SA Pelegrino, Eugene A Volpe, Stephen C Pflugfelder, Cintia S de Paiva

**Affiliations:** Ocular Surface Center, Department of Ophthalmology, Cullen Eye Institute, Baylor College of Medicine, 6565 Fannin Street, NC505G, Houston, TX 77030 Texas USA

## Abstract

**Introduction:**

The lacrimal gland (LG) of the CD25^-^/^-^ model of Sjögren’s syndrome (SS) has high interleukin (IL)-17, IL-13 and interferon-gamma (IFN-γ) cytokines. The specific contribution of these cytokines to the onset and severity of dacryoadenitis in the CD25^-^/^-^ mice has not been evaluated.

**Methods:**

CD25^−^/^−^IL-17A^−^/^−^, CD25^−^/^−^IL-17^−^/^−^IFN-γ^−^/^−^ and CD25^−^/^−^IFN-γ^−^/^−^ were used at 4, 8, 12, 16 weeks (W). Total lymphocytic infiltration was evaluated by histology and characterized by flow cytometry. Epidermal growth factor (EGF) concentration was measured in tears. Immunofluorescent staining evaluated expression of IFN-γ receptor (IFN-γR) and apoptosis. Real-time PCR evaluated inflammatory and T cell-related cytokines expression in LG. Caspase-3, -8, -9 activities was assayed in LG lysates. T helper cytokines were measured in serum by Luminex assay.

**Results:**

The greatest total LG infiltration at 8 W was seen in CD25^−^/^−^IL-17A^−^/^−^ (95%), followed by CD25^−^/^−^ (71%) and IL-17^−^/^−^ (12%). Tear EGF concentration was in normal range in CD25^−^/^−^ at 4 W and in very low levels in both CD25^−^/^−^ and CD25^−^/^−^IL-17A^−^/^−^. CD25^−^/^−^ had high levels of inflammatory cytokines transcripts in LG compared to IL-17^−^/^−^ mice; however, CD25^−^/^−^IL-17A^−^/^−^ had even higher IL-1β, IFN-γR, caspase-3, -8, -9 mRNA levels, greater immunoreactivity to IFN-γR in LG acini, greater number of apoptotic^+^ cells and greater caspases activities in the LG at 8 W. CD25^−^/^−^IL-17A^−^/^−^ had lower IL-13 concentration and lower IL-13/IFN-γ ratio compared to CD25^−^/^−^ in serum. CD25^−^/^−^IFN-γ^−^/^−^ had lower number of apoptotic^+^ cells and decreased caspase-3 expression in LG. CD25^−^/^−^IL-17^−^/^−^IFN-γ^−^/^−^ had lower total lymphocytic cell infiltration at 8 W (48%), CD4^+^T cell infiltration and expression of IFN-γR and apoptotic^+^ cells in the LG and increased tear EGF concentration in tears.

**Conclusions:**

IFN-γ is critical for LG destruction and secretory dysfunction in the CD25^−^/^−^ model of SS. Altered balance between IFN-γ and IL-13 in the CD25^−^/^−^IL-17A^−^/^−^ mice accelerates LG destruction by increasing glandular apoptosis and facilitating apoptosis through increased expression of IFN-γR by glandular epithelium and activation of caspases. Targeting both IFN-γ and IL-17 may be beneficial for treating the LG inflammation in SS.

## Introduction

Sjögren’s syndrome (SS) is an autoimmune disorder characterized by progressive lymphocytic infiltration of the salivary and lacrimal gland (LG) leading to dry eye and dry mouth. Glandular infiltrates are composed of a mix of dendritic cells, macrophages, CD4^+^, CD8^+^, natural killer (NK), Foxp3^+^, B cells, dendritic cells [1]. Despite expanding efforts to define the immunopathology of SS, the underlying molecular mechanisms responsible for the impaired secretory function of the inflamed LG remain incompletely understood, as several molecules have been known to impair secretion in *in vitro* models, including interleukin (IL) IL-1, IL-6, nitric oxide (NO), anti-muscarinic receptor, anti-Ro immunoglobulin G (IgG) [2-5]. Mouse models that recapitulate some features of SS have been used to study the pathogenesis, including the IL-2 receptor alpha chain (CD25) knockout (KO) [6,7].

IL-2 signals through its heterodimer receptor composed of three individual chains. CD25 binds IL-2 with high affinity and it is considered its main receptor. Mice lacking CD25 have phenotype similar to mice lacking IL-2 itself [8,9]. As IL-2 is responsible for expansion and differentiation of T regulatory cells (Tregs) and also activation-induced cell death, autoimmunity arises in both IL-2^−/−^ and CD25^−/−^ strains [6,8,10]. The CD25KO mouse develops spontaneous multiorgan inflammatory disease, inclusive of exocrine glands and gastrointestinal tract, and a profound hemolytic anemia that leads to early mortality. The spontaneous dacryoadenitis that develops in these mice is age dependent, with 50% of the LG infiltrated by 8 weeks (W), evolving to complete atrophy and periductal fibrosis at age 16 W [7]. Our previous studies with this mouse strain have found elevated IL-1β, IL-17 and interferon-gamma (IFN-γ) transcripts in the LG [7]. We have shown that IL-17A levels in the LG peak at 8 to 12 W with a steady decline after, suggesting that IL-17 could be involved in earlier disease development [7,11]. Similar to findings in the nonobese diabetic (NOD) strain [12], deletion of IFN-γ in the CD25KO delays onset and severity of dacryoadenitis, but does not prevent development of lymphocytic infiltration [13].

IL-17-producing T lymphocytes have been recently shown to comprise a T helper (Th) lineage known as Th17 cells, which are distinct from Th1 and Th2 cells. Th1 cells contribute to host defense of viral, fungal, and intracellular bacterial infections, and are characterized by the production of IFN-γ. Th17 cells have been shown to be involved in the pathogenesis of many autoimmune and inflammatory diseases, inclusive of SS.

IL-13 regulate IFN-γ both *in vitro* and *in vivo* [14]. In murine models of allergic asthma, mice repeatedly exposed to allergens or IL-13 develop goblet cell hyperplasia of the airway epithelium [15-17]. IL-13 has also antiapoptotic effects on airway and colonic epithelia, two tissues that are rich in goblet cells [18,19]. IL-13 is expressed by Th2 cells and plays a role in B cell activation.

The specific role of IL-17, IL-13 and IFN-γ in the inflammation that develops in the CD25KO has not been evaluated. To accomplish this, we used several double knockouts (DKO), including a CD25/IL-17 DKO, a CD25/IFN-γ DKO as well as a CD25/IL-17/IFN-γ triple KO (TKO) mouse. Herein we describe that deletion of IL-17 in the CD25KO model accelerates LG lymphocytic infiltration and acini apoptosis, due to a high IFN-γ receptor, high tumor necrosis factor (TNF)-related apoptosis-inducing ligand (TRAIL), Fas-ligand (Fas-L) and high caspase-3 and -9 and low local IL-13/IFN-γ ratio, while the deletion of IFN-γ decreases caspase activity levels and number of terminal deoxynucleotidyl transferase dUTP nick end labeling (TUNEL)-positive (+) cells. Deletion of IL-17A and IFN-γ in the CD25KO (creating a CD25/IL-17/IFN-γ TKO) ameliorates dacryoadenitis and improves glandular function, demonstrating that IFN-γ is key to the autoimmunity in this model.

## Methods

### Animals

This research protocol was approved by the Baylor College of Medicine Center for Comparative Medicine, and it conformed to the standards in the ARVO Statement for Use of Animals in Ophthalmic and Vision Research.

CD25^+/−^ (B6.129S4-*IL-2ra*^*tm1Dw*^/J), IFN-γKO and C57BL/6 J mice breeding pairs were purchased from Jackson Laboratories (Bar Harbor, ME, USA) for establishing of breeder colonies. IL-17KO mice were kindly provided by Dr. Yoichiro Iwakura [20].

To create a CD25/IL-17 double KO (CD25^−/−^IL-17^−/−^; CD25/IL-17 DKO), IL-17KO mice were mated with CD25^+/−^ mice. F1 was genotyped and CD25^+/−^IL-17^+/−^ mice were mated. F2 offspring were genotyped. CD25^−/−^IL-17^−/−^ were obtained after interbreeding of CD25^+/−^IL-17^−/−^ mice. The genotype of KO strains was confirmed using a previously reported protocol. Mice were used at 4, 8, 12, 16 W. A minimum of 24 animals per age (4, 8, 12, 16 W) per strain (CD25KO, IL-17KO and CD25/IL-17 DKO) were used: five (histology), six (flow cytometry), eight (gene expression), five (caspase activity). Tear collection was performed when mice were alive (n = 12). In some strains/age, up to 20 animals were used for flow cytometry and up to eight animals were used for caspase activity.

To create a CD25/IL-17/IFN-γ triple TKO (CD25^−/−^IL-17^−/−^IFN-γ^−/−^), CD25^+/−^IL-17^−/−^ were mated with CD25^+/−^IFN-γ^−/−^ mice. F1 was genotyped and CD25^+/−^IL-17^+/−^IFN-γ^+/−^ were mated again. F2 offspring was genotyped and CD25^+/−^IL-17^−/−^IFN-γ^−/−^ (IL-17/IFN-γ DKO) were used as breeders to generate CD25/IL-17/IFN-γ TKO mice. The IL-17/IFN-γ DKO bred very slowly and very few CD25/IL-17/IFN-γ TKO mice were generated. The genotype of KO strains was confirmed using a previously reported protocol. CD25/IL-17/IFN-γ TKO and IL-17/IFN-γ DKO mice were used at 8, 12, and 16 W: five (histology), twelve (flow cytometry). An additional six (4 W) and twelve C57BL/6 mice (six for 4 and 8 W) were used for histology, tear epidermal growth factor (EGF) assay and serum collection.

To create a CD25/IFN-γ DKO (CD25^−/−^IFN-γ^−/−^), IFN-γKO mice were mated with CD25^+/−^ mice. F1 was genotyped and CD25^+/−^IFN-γ^+/−^ mice were mated. F2 offspring were genotyped. CD25^−/−^IFN-γ^−/−^ were obtained after interbreeding of CD25^+/−^IFN-γ^−/−^ mice as previously described [13]. CD25/IFN-γ DKO were used at 4 and 8 W: four animals for histology and four animals for gene expression.

### Histology and measurement of total infiltration

Extraorbital LG were excised, fixed in 10% formalin, paraffin embedded, and 8-μm sections were cut as previously described [13]. Sections were stained with hematoxylin and eosin (H&E) for evaluating morphology. The area of lymphocytic infiltration was circumscribed in digital images of H&E-stained sections as previously described [7]. The percentage infiltration was calculated as area of infiltration × 100/total area.

### Immunohistochemistry (IHC) and laser confocal microscopy

Extraorbital LG from each strain/age (n = 5) were excised, embedded in optimal cutting temperature compound (VWR, Suwanee, GA, USA), and flash frozen in liquid nitrogen and stored at -80c.

IHC in LG cryosections was performed by using CD4 (BD Pharmingen, San Jose, CA, USA; clone H129.9, 10 μg/ml), CD8a (BD Pharmingen; clone 53e6.7, 3.125 μg/ml), or CD19 (Abcam, Cambridge, MA, USA; clone 6D5, 2 μg/ml) antibodies. Staining was performed with appropriate biotinylated secondary antibodies (all from BD Pharmingen) and a Vectastain Elite ABC kit with Nova Red reagents (Vector Laboratories, Burlingame, CA, USA). Secondary antibody alone and appropriate anti-mouse isotype (BD Biosciences, San Diego, CA, USA) were used as controls. Six sections from each animal/group/time point were examined and photographed with a microscope equipped with a digital camera (Eclipse E400 with a DS-Fi1; Nikon, Melville, NY, USA).

Immunofluorescent staining in LG cryosections was performed by using primary antibodies against Fas-L or IFN-γ receptor (1:100 for both, sc-834 and sc-703, respectively; Santa Cruz Biotechnology, Dallas, TX, USA). The tissue sections (two slides per animal) for immunofluorescent staining were fixed in acetone at −20°C for 10 minutes, blocked in 20% normal goat serum in phosphate-buffered saline (PBS) for 60 minutes and primary antibodies were applied and incubated for 1 hour at room temperature (RT). Secondary antibody, Alexa-Fluor 488-conjugated goat anti-rabbit IgG (1:100 dilution) were applied, sections was incubated in a dark chamber for 1 hour, followed by counterstaining with propidium iodide (PI; 2 μg/mL in PBS) for 5 minutes. Secondary antibody alone was also performed as negative controls.

Digital confocal images (512 × 512 pixels) were captured with a laser scanning confocal microscope (LSM 510, with krypton-argon and He-Ne laser; Carl Zeiss Meditec, Inc., Thornwood, NY, USA) with 488-nm excitation and 543-nm emission filters (LP505 and LP560, respectively; Carl Zeiss Meditec, Inc.) and were acquired with a 40/1.3× oil-immersion objective. Images from all samples were captured with identical photomultiplier tube gain settings and were processed with the microscope system (LSM-PC software; Carl Zeiss Meditec, Inc.).

### Flow cytometry analysis of infiltrating cells

Single-cell suspensions of LG from all strains were prepared and stained for CD4, CD8 and B220 surface markers as previously reported [13]. The initial leukocyte gate in flow cytometry analysis was confirmed to have greater than 98% of CD45^+^ cells.

### RNA isolation and quantitative PCR

Extraorbital LGs from IL-17KO, CD25KO, CD25/IL-17 DKO, and C57BL/6 were excised and total RNA was extracted and processed as previously described [13]. Eight samples per strain/age were used, and one sample consisted of pooled glands from the same animal. First-strand cDNA was synthesized.

Quantitative real-time PCR was performed with specific minor groove binder (MGB) probes as previously published [21]. Murine MGB probes were IFN-γ (Mm00801778), IL-13 (Mm00434165), IL-4 (Mm00445259), IL-5 (Mm99999063), hypoxanthine phosphoribosyltransferase (HPRT1) (Mm00446968), caspase-3 (Mm00438045), caspase-8 (Mm00802247), caspase-9 (Mm01348848), major histocompatibility complex class II (MHC-II) (Mm00482914) TNF-α (Mm99999068), IL-1β (Mm00434228), IL-17 (Mm00439618), IFN-γ receptor (Mm00599890), TRAIL (Mm01283606) and Fas-L (Mm00438864). The HPRT-1 gene was used as an endogenous reference for each reaction. The results of real time PCR were analyzed by the comparative CT method and the results were normalized by the CT value of HPRT-1. The mean CT of relative mRNA level in the 4-W IL-17KO group was used as the calibrator.

### Tear washings and EGF enzyme-linked immunosorbent assay (ELISA)

ELISA was performed to measure EGF concentrations in tear fluid washings using a commercial ELISA kit (R&D Systems, Minneapolis, MN, USA) as previously described [13]. Tear fluid washings were collected from 12 animals/strain/age [13]. One sample consisted of tear washings from both eyes of one mouse pooled (2 μL) in PBS + 0.1% bovine serum albumin (BSA) (8 μL) and stored at −80°C until the assay was performed. Results are presented as mean ± standard deviation (SD) (pg/ml).

### Caspase-3, −8, and −9 activation fluorometric assays

The activation of caspase-3, -8, and -9 was measured in LG lysates according to the manufacturer’s protocol (K105-200, K112-200, and K118-200, respectively; BioVision, Inc., Mountain View, CA, USA). Protein concentration was measured using a micro BSA protein assay kit (Thermo Fisher Scientific, Waltham, MA, USA). Five to eight samples per age/strain were used. Caspase-3, -8, and -9 activities were measured (50 μg/sample) by following the cleavage of the fluorescent substrate analogs in a fluorescent plate reader (Tecan Infinite M200, Magellan V6.55 software; Tecan, Männedorf, Switzerland) with 400-nm excitation filter and 505-nm emission filter. The results were exported and averaged.

### TUNEL assay

The TUNEL assay was performed with a kit (ApopTag; Intergen Co., Purchase, NY, USA), in cryosections of LG (n = 3/strain/age), as previously described [22]. TUNEL^+^ cells were counted in digital images at 20X within each group and results averaged.

### IL-4, IL-5, IL-13, IL-17 and IFN-γ LUMINEX assay in serum

Concentrations of IL-4, IL-5, IL-13, IL-17 and IFN-γ in serum were measured using a multiplex Mouse Cytokine Magnetic Bead Panel (Millipex Map Kit; EMD Millipore Corp, Billerica, MA, USA). Serum was collected from mice (n = 4 to 8/strain) through cardiac puncture immediately after euthanasia and frozen at −80°C until ready to use. A 25 μl/sample was used according to the manufacturer’s protocol. Labeled cytokines were read by a Luminex 100 with xPONENT 3.1 (Luminex, Austin, TX, USA). At least 50 events per bead were read and the data were analyzed using Millipex Analyst software (EMD Millipore).

### Statistical analysis

Two-way analysis of variance (ANOVA) or Kruskall-Wallis test with Tukey’s *post hoc* testing was used for statistical comparisons with alpha of 0.05. These tests were performed using GraphPad Prism 6.0 software (GraphPad Software Inc., San Diego, CA, USA).

## Results

### Deletion of IL-17A in the CD25KO worsen dacryoadenitis

**Figure 1:**
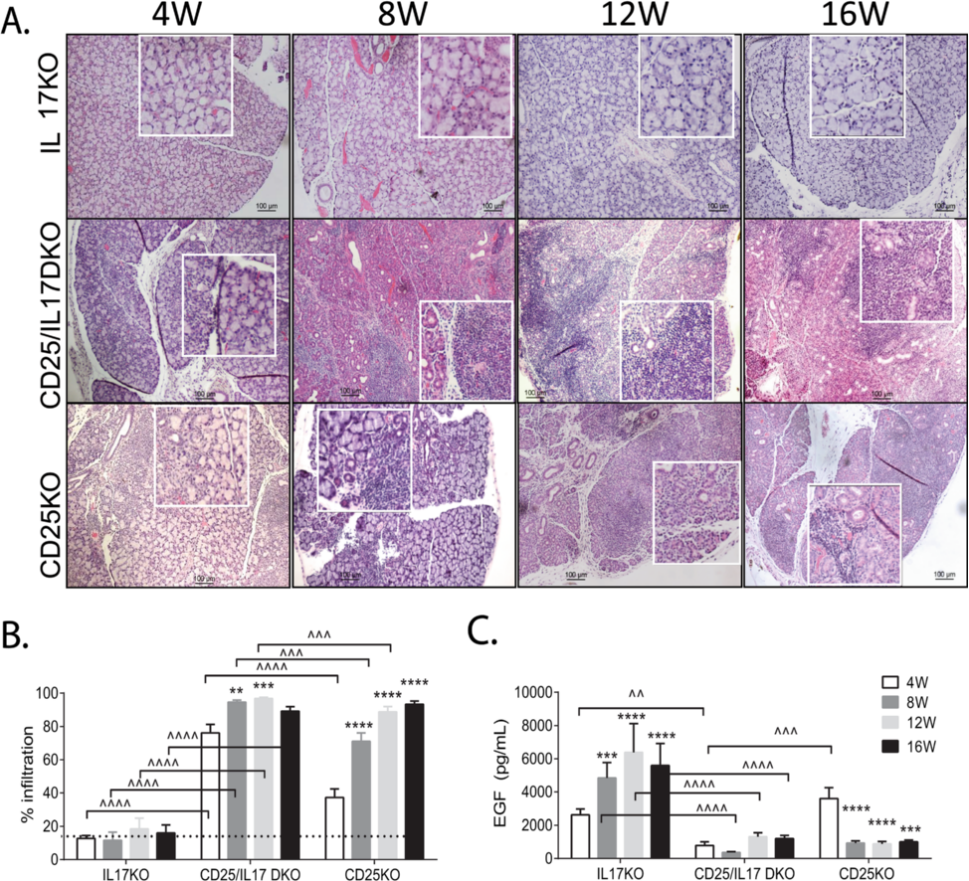
**Accelerated and more severe dacryoadenitis in CD25/IL-17 DKO LG. (A)** Representative images of H&E-stained LG from mice aged 4 to 16 weeks (W). The square is a higher magnification of the area underneath. Scale bar = 100 μm. **(B)** Percent area of lymphocytic infiltration measured in digital images of H&E-stained LG sections from IL-17KO, CD25/IL-17 DKO and CD25KO mice at 4, 8, 12, and 16 W. **(C)** EGF concentration measured in tear washings from different strains. ^*^ *P* <0.05;^**^ *P* <0.01, ^***^ *P* <0.001; ^****^ *P* <0.0001 within strain comparison vs. 4 W. ^^^ *P* <0.05; ^^^^ *P* <0.01, ^^^^^ *P* <0.001; ^^^^^^ *P* <0.0001 interstrain comparison. CD25/IFN-γ DKO, CD25^−/−^IFN-γ^−/−^; CD25KO, CD25 knockout; DKO, double knockout; EGF, epidermal growth factor; H&E, hematoxylin and eosin; IL, interleukin; IL-17KO, IL-17 knockout; KO, knockout; LG, lacrimal gland.

Our previous studies showed that CD25KO mice have increased expression of both IL-17A and IFN-γ transcripts in LG tissue [7] and that deletion of IFN-γ delayed the onset and severity of dacryoadenitis in this strain [13]. To dissect the specific contribution of IL-17A in this model, we compared CD25KO and CD25/IL-17 DKO mice with age-matched IL-17KO mice. Neither the CD25KO [13], nor the IL-17KO strain have a gender bias (data not shown). No gender difference in severity of LG infiltration in the CD25/IL-17 DKO or CD25/IL-17/IFN-γ TKO strain was observed; therefore, the data represent an average of both genders (1:1) for each parameter/age. Increased mortality was seen in CD25/IL-17 DKO strain compared to CD25KO strain, as early as 5 W, with few mice reaching 16 W of age. Sparse cell infiltration was observed around the ducts in IL-17KO LG, while both CD25KO and CD25/IL-17 DKO showed increased lymphocytic infiltration (approximately 40% and approximately 80% respectively, Figure 1A, B) at 4 W. After 8 W, both CD25KO and CD25/IL-17 DKO had similar total acinar loss, ductal proliferation, which progressed to atrophy by 16 W. Normal acini were seen in CD25/IL-17 DKO LG only at 4 W (Figure 1A, B).

As a measurement of LG secretory function, EGF concentration in tears was measured as previously described [13]. A progressive increase in EGF concentration in tears of IL-17KO mice was seen as they aged, suggesting normal maturation and function of LG epithelia (Figure 1C). Both CD25KO and CD25/IL-17 DKO had significantly lower EGF concentration compared to IL-17KO mice at all ages.

**Figure 2:**
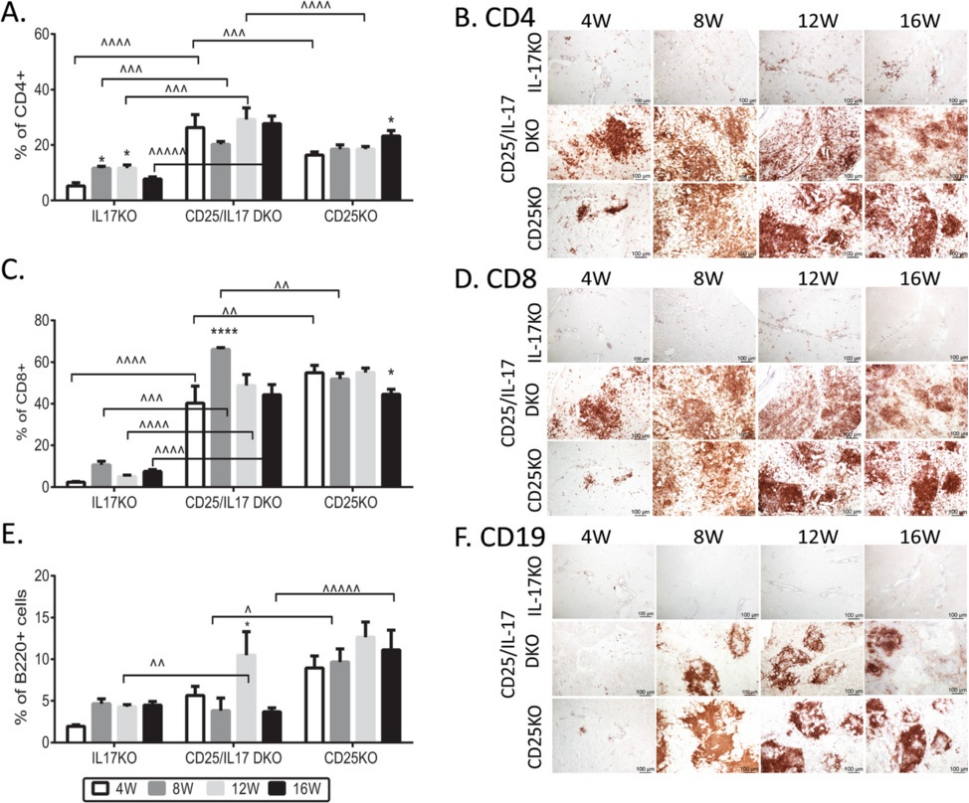
**Lacrimal gland infiltrates. (A, C, E)** Percentage of CD4^+^ T **(A)**; CD8^+^ **(C)** and B220^+^ cells **(E)** gated by flow cytometry of LG cells from IL-17KO, CD25KO, CD25/IL-17 DKO mice aged 4 to 16 weeks of age (W). Data are presented as mean ± SEM of six to twelve individual samples/strain/age. **(B, D, F)** Representative images of immunohistochemistry of LG sections stained for CD4 **(B)**, CD8 **(D)** and CD19 **(F)** positive cells (stained in red) in sections obtained from all three strains. Scale bar = 100 μm. ^*^ *P* <0.05; ^**^ *P* <0.01, ^***^ *P* <0.001; ^****^ *P* <0.0001 within strain comparison vs. 4 W. ^^^ *P* <0.05; ^^^^ *P* <0.01, ^^^^^ *P* <0.001; ^^^^^^ *P* <0.0001 interstrain comparison. CD25/IFN-γ DKO, CD25^−/−^IFN-γ^−/−^; CD25KO, CD25 knockout; DKO, double knockout; IL, interleukin; IL-17KO, IL-17 knockout; LG, lacrimal gland; SEM, standard error of the mean.

CD25/IL-17 DKO showed a significantly greater percentage of CD4^+^ cells than the CD25KO strain at 4 and 12 W (Figure 2A). Flow cytometry showed a predominance of CD8^+^ T cells over CD4^+^ T cells in CD25KO and CD25/IL-17 DKO LGs at all ages compared to IL-17KO mice (Figure 2C). CD4 and CD8^+^ T cells were mainly seen around ducts (Figure 2B, D). CD25KO had the greatest percentage of B220^+^ cells compared to IL-17KO (at all ages) and higher percentage than CD25/IL-17 DKO (Figure 2E) at 8 and 12 W. Minimal CD19^+^ B cell infiltration was observed in IL-17KO at all ages (Figure 2F). Sparse infiltrating CD19^+^ cells were seen in CD25/IL-17 DKO and CD25KO at 4 W whereas they were easily identified as these mice aged (Figure 2F). Two types of CD19^+^ B cell infiltration were observed: the first resembled IL-17KO LGs, where B cells were dispersed throughout the gland and did not accumulate in foci, while the second was suggestive of germinal center formation within the LG (Figure 2F, aged 8 to 16 W).

Taken together, these results show that deletion of IL-17A in the CD25KO model of SS accelerated LG destruction and promoted progressive increased lymphocytic infiltration.

### Increased inflammation and Th-related cytokines in CD25/IL-17 DKO LG

**Figure 3:**
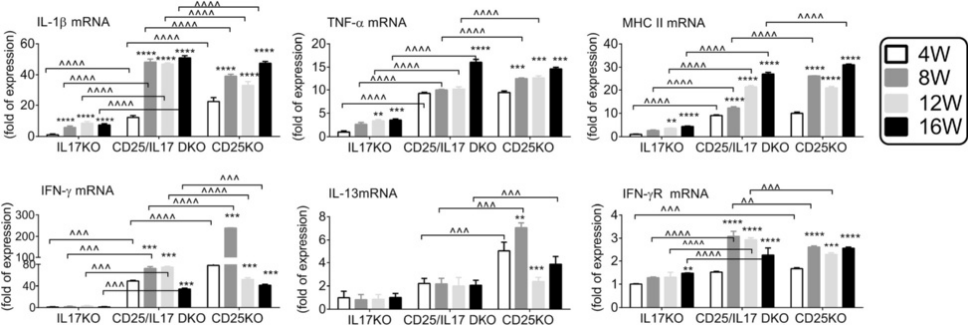
**Comparison of inflammatory markers and T cell-related cytokines in LG of knockouts.** Relative fold of expression of IL-1β, TNF-α, MHC II, IFN-γ, IL-13 and IFN-γ receptor. Data are presented as mean ± SEM of eight individual samples/strain/age. ^*^ *P* <0.05; ^**^ *P* <0.01, ^***^ *P* <0.001; ^****^ *P* <0.0001 within strain comparison vs. 4 weeks. ^^^ *P* <0.05; ^^^^ *P* <0.01, ^^^^^ *P* <0.001; ^^^^^^ *P* <0.0001 interstrain comparison. IFN-γ, interferon-gamma; IL, interleukin; LG, lacrimal gland; MHC-II, major histocompatibility complex class II; SEM, standard error of the mean TNF-α, tumor necrosis factor alpha. R = receptor.

To investigate the phenotype of infiltrating cells, we evaluated the expression of MHC II, inflammatory cytokines (IL-1β, TNF-α), IL-13, IFN-γ, IFN-γ receptor (IFN-γR) and IL-13 using the 4 W IL-17KO LG as calibrator control, since this control strain had minimal T cell infiltration (Figure 3). Real-time PCR analysis showed that IL-1β, TNF-α, IFN-γ and MHC II were all significantly elevated in CD25/IL-17 DKO and CD25KO compared to IL-17KO LG at all ages, although aging from 4 to 16 W significantly increased levels of IL-1β, TNF-α and MHC II transcripts in IL-17KO mice.

Expression of IL-1β mRNA was higher in CD25KO at 4 W, and expression of IFN-γ mRNA was higher at both 4 W and 8 W. By 8 W and 12 W, expression of both transcripts was higher in CD25/IL-17 DKO.

Although the level of TNF-α transcripts was similar in CD25KO and CD25/IL-17 DKO at 4 W, it increased steadily in CD25KO from 4 to 12 W, while in the CD25/IL-17 DKO it did not change until 16 W, when it was significantly higher than CD25KO.

Interestingly, MHC II expression in the CD25KO had a bimodal distribution peaking at 8 W (approximately 27-fold) and 16 W (31-fold), while in the CD25/IL-17 DKO it increased gradually, reaching the same level as the CD25KO at 12 W.

IL-13 has been described to be antiapoptotic in colonic and airway epithelial cells as well as conjunctival fibroblasts [18,19,23]. Expression of IL-13 mRNA in the CD25KO increased over a biphasic time course. Expression in CD25/IL-17 DKO was markedly suppressed compared to CD25KO at 4 W, 8 W, and 16 W (Figure 3). IL-4 and IL-5 transcripts were barely detected in some samples and were undetected in many samples.

Our results indicate that CD25/1L-17 DKO mice have greater expression of IL-1β, while lower levels of IL-13 than CD25KO, but both strains had increased TNF-α and MHC II compared to IL-17KO. CD25KO LG have increased IL-17A, IFN-γ and IL-13, while CD25/IL-17 DKO mice have only elevated IFN-γ. IL-13 may have a protective role counteracting IFN-γ, as higher levels of IL-13 transcripts were seen in CD25KO mice, which had less severe disease than the CD25/IL-17 DKO.

### Systemic Th2 cytokines are suppressed in the CD25/IL-17 DKO compared to the CD25KO

**Figure 4:**
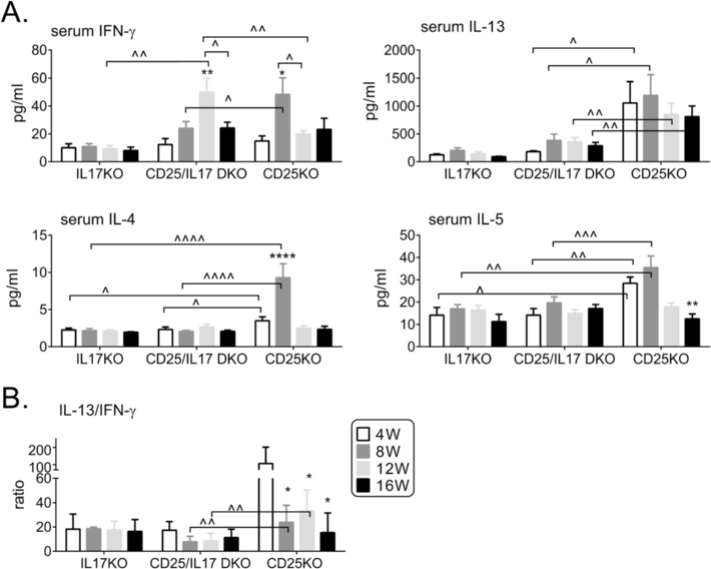
**Comparison of T helper cell-related cytokines in serum of knockouts. (A)** Concentration of IFN-γ, IL-13, IL-4 and IL-5 in serum from IL-17KO, CD25/IL-17 DKO, CD25KO mice. Data are presented as mean ± SEM of eight individual strains/ages. **(B)** Bar graph showing mean ± SEM of IL-13/IFN-γ ratio of serum from IL-17KO, CD25/IL-17 DKO, CD25KO mice. ^*^ *P* <0.05; ^**^ *P* <0.01, ^***^ *P* <0.001; ^****^ *P* <0.0001 within strain comparison vs. 4 weeks. ^^^ *P* <0.05; ^^^^ *P* <0.01, ^^^^^ *P* <0.001; ^^^^^^ *P* <0.0001 interstrain comparison. CD25/IFN-γ DKO, CD25^−/−^IFN-γ^−/−^; CD25KO, CD25 knockout; DKO, double knockout; IFN-γ, interferon-gamma; IL, interleukin; IL-17KO, IL-17 knockout; LG, lacrimal gland; SEM, standard error of the mean; W, weeks.

IL-17A serum levels were 10-fold higher in CD25KO mice than C57BL/6 mice at 8 W (78.82 ± 48 vs. 7.57 ± 4.04 pg/mL, respectively, *P* = 0.001). A progressive systemic increase in IFN-γ was measured in serum of CD25/IL-17 DKO mice as they aged from 4 to 12 W (Figure 4A). IL-13 levels in serum mirrored gene expression in the LG as they were significantly elevated in CD25KO at all ages compared to CD25/IL-17 DKO (Figure 4A). IL-4 and IL-5 levels in serum of CD25KO were significantly increased in CD25KO at 4 and 8 W compared to CD25/IL-17 DKO and low levels were measured in IL-17KO at all ages (Figure 4A). The IL-13/IFN-γ ratio in sera using the mean values for all three strains showed that CD25KO have a higher IL-13 systemic response than CD25/IL-17 DKO (Figure 4B). The IL-4/IFN-γ and IL-5/IFN-γ ratio in CD25/IL-17 DKO was also lower compared to CD25KO mice (data not shown).

Taken together, these results showed that CD25KO mice have high levels of systemic Th1, Th17 and Th2 signature cytokines but an unchecked systemic elevated Th1 response (with low levels of Th2) is observed with aging in the CD25/IL-17 DKO mice.

### Role of IL-17 and IFN-γ in the LG apoptosis in CD25KO mice

**Figure 5:**
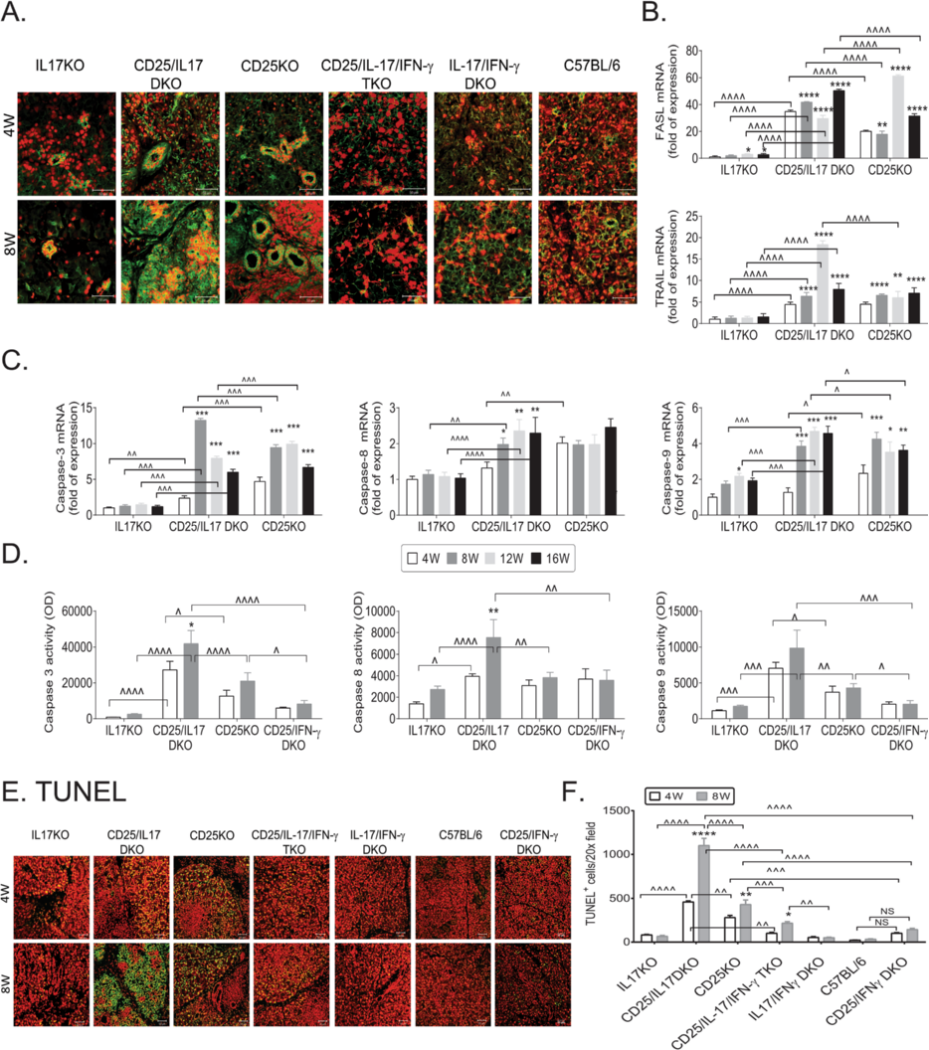
**Comparison of apoptosis among different double knockouts. (A)** Representative merged images of laser scanning immunofluorescent confocal micrographs of LG immunostained for IFN-γ receptor (IFN-γR, green) with propidium idodide counterstaining (red) in IL-17KO, CD25KO, CD25/IL-17 DKO, CD25/IL-17/IFN-γ TKO, IL-17/IFN-γ DKO and C57BL/6. Scale bar = 50 μm. **(B-C)** Relative fold of expression of Fas-L and TRAIL **(B)** and caspase-3, -8 and -9 **(C)** in LG lysates of IL-17KO, CD25/IL-17 DKO and CD25KO mice aged 4 to 8 weeks (W). Data are presented as mean ± SEM of eight individual samples/strains/ages. **(D)** Caspase-3, -8 and -9 activity assay results from LG lysates of IL-17KO, CD25/IL-17 DKO, CD25KO and CD25/IFN-γ DKO mice aged 4 to 8 W. Data are presented as mean ± SEM of five to eight individual samples/strains/ages. **(E)** Representative merged images of laser scanning fluorescent confocal micrographs of TUNEL-stained LG (green) with propidium iodide counterstaining (red) in IL-17KO, CD25KO, CD25/IL-17 DKO, CD25/IL-17/IFN-γ TKO, IL-17/IFN-γ DKO, C57BL/6 and CD25/IFN-γ DKO mice used to generate the bar graphs in **(F)** Scale bar = 50 μm. F. TUNEL^+^ cells (mean ± standard deviation (SD)) among the different groups. ^*^ *P* <0.05; ^**^ *P* <0.01, ^***^ *P* <0.001; ^****^ *P* <0.0001 within strain comparison vs. 4 W. ^^^ *P* <0.05; ^^^^ *P* <0.01, ^^^^^ *P* <0.001; ^^^^^^ *P* <0.0001 interstrain comparison. CD25/IFN-γ DKO, CD25^−/−^IFN-γ^−/−^; CD25/IL-17/IFN-γ TKO, CD25^−/−^IL-17^−/−^IFN-γ−/−; CD25KO, CD25 knockout; DKO, double knockout; Fas-L, Fas-ligand; IFN-γ, interferon-gamma; IL-17/IFN-γ DKO, CD25^+/−^IL-17^−/−^IFN-γ^−/−^; IL, interleukin; IL-17KO, IL-17 knockout; LG, lacrimal gland; SEM, standard error of the mean; TKO, triple knockout; TNF, tumor necrosis factor; TRAIL, TNF-related apoptosis-inducing ligand; TUNEL, terminal deoxynucleotidyl transferase dUTP nick end labeling; W, weeks.

We have previously demonstrated a proapoptotic role of IFN-γ in our inducible dry eye model, as antibody neutralization of IFN-γ prevented desiccation-induced goblet cell loss [24]. Because we found high IFN-γ and low IL-13 transcripts in CD25/IL-17 DKO mice, we hypothesize that these mice might be more susceptible to the apoptotic effects of IFN-γ due to its lower IL-13 levels, since IL-13 has been shown to have antiapoptotic effects and to downregulate IFN-γR expression [18,19]. To address this, we investigated IFN-γR expression by real-time PCR and immunostaining. Both CD25KO and CD25/IL-17 DKO had higher expression of IFN-γR at 4 W compared to IL-17KO; but greater mRNA levels were observed in CD25/IL-17 DKO LG compared to CD25KO at 8 and 12 W (Figure 5A). Immunostaining evaluated protein expression at 4 and 8 W because these ages had the greatest differences. Minimal immunoreactivity for IFN-γR was present in IL-17KO and C57BL/6 (Figure 5A), while increased immunostaining was seen in the LG ducts in both CD25KO and CD25/IL-17 DKO at 4 W, which increased as these mice aged to 8 W.

Since expression of Fas-L on infiltrating lymphocytes has been shown to cause apoptosis to surrounding cells [25-27], we investigated the expression of Fas-L and TRAIL. Fas-L was significantly increased in both CD25KO and CD25/IL-17DKO compared to IL-17KO, and IL-17 DKO had significantly higher levels of Fas-L at 4, 8 and 16 W compared to CD25KO.

There was an age-progressive increase in TRAIL mRNA levels in CD25/IL-17 DKO LG from 4 to 8 W and it was significantly higher than CD25KO LG at 12 W (Figure 5B).

Because Fas-L and IFN-γ-mediated apoptosis ultimately involves activation of caspases [24,28], we investigated apoptotic pathway mediators in LG lysates. Caspase-3 is the final common apoptosis pathway, while caspase-9 is involved in the intrinsic pathway and caspase-8 is involved in the extrinsic one. Caspase-3, -8, -9 mRNA levels were elevated in CD25/IL-17 DKO and CD25KO compared to IL-17KO at 8, 12 and 16 W (Figure 5C). A progressive increase in caspase-3 and -8 transcripts expression was seen in CD25/IL-17 DKO LG as these mice aged. Significantly higher levels of caspase-3, -8 and -9 were seen in CD25KO at 4 W compared to the CD25/IL-17 DKO. Caspase-3 transcripts were significantly upregulated in CD25/IL-17 DKO, while caspase-8 and -9 were equally elevated in both CD25KO and CD25/IL-17 DKO at 8 W. Results from gene expression were confirmed by caspase activity assays. IL-17KO had the lowest caspase activity levels among all three strains, corroborating the PCR results. Compared to CD25KO mice, CD25/IL-17 DKO LG had significantly higher caspase-3, and -9 activity levels at 4 W and significantly higher caspase-3, -8 and -9 activity levels at 8 W (Figure 5D). An increase with aging from 4 to 8 W within the CD25/IL-17 DKO LG was observed for caspase-3 and -8.

TUNEL detects fragmented DNA, another sign of apoptosis. Increased TUNEL^+^ cells were seen in LG sections of both CD25/IL-17 DKO and CD25KO at 4 W compared to IL-17KO LG control (459 ± 16.72 and 281 ± 14.29 vs. 81.67 ± 7.56 TUNEL^+^ cells/20X field, *P* <0.0001 for both, respectively, Figure 5E and F). TUNEL^+^ cells were seen in the glandular epithelial cells, but not in the infiltrating cells (Figure 5E). At 8 W, a further increase in TUNEL^+^ cells was seen in the CD25/IL-17 DKO mice compared to its baseline (1,100.67 ± 101.85 vs. 459 ± 16.72 TUNEL^+^ cells/20X field, *P* <0.0001).

These results suggest that increased IFN-γR expression in the CD25/IL-17 DKO mice sensitizes them to IFN-γ-induced apoptosis and these changes may be potentiated in the low antiapoptotic IL-13 environment of this strain compared to CD25KO.

### Deletion of IFN-γ in CD25KO decreases apoptosis

We have shown that antibody neutralization of IFN-γ prevented desiccation-induced increase in caspase-3 mRNA and immunoreactivity while exogenous administration of IFN-γ during desiccating stress induced caspase-9 mRNA and activity [24,29]. To confirm the role of IFN-γ in promoting glandular apoptosis in the CD25KO model, we evaluated caspase activity in LG protein lysates and performed TUNEL assay in CD25/IFN-γ DKO LG cryosections at 4 W and 8 W. These mice have significantly delayed and less severe dacryoadenitis compared to CD25KO mice at 8 W [10]. We observed significantly lower caspase-3 and -9 activity levels in CD25/IFN-γ DKO LG compared to CD25KO at 8 W and CD25/IL-17 DKO (Figure 5D). Although the level of caspase-8 activity was significantly lower than the CD25/IL-17 DKO, it was not different from CD25KO mice (Figure 5D).

CD25/IFN-γ DKO at 4 and 8 W had very few TUNEL^+^ cells, and they were significantly lower than CD25KO parental strain (101 ± 14 and 143 ± 17.44 vs. 281 ± 14.29 and 429 ± 23.24 TUNEL^+^ cells/20X field, *P* <0.001 and *P* <0.0001, respectively, Figure 5E and F). These results confirm the pathogenic role of IFN-γ in inducing glandular apoptosis through intrinsic apoptosis pathway (caspase-9) as deletion of IFN-γ in the CD25KO model significantly decreases caspase activity levels and TUNEL^+^ cells.

### Deletion of IFN-γ in CD25/IL-17 DKO ameliorates SS-like disease

Next we investigated if deletion of IFN-γ in the CD25/IL-17 DKO mouse would diminish LG disease by generating a CD25/IL-17/IFN-γ TKO mouse (CD25^−/−^IL-17^−/−^IFN-γ^−/−^) and compared to IL-17/IFN-γ DKO littermates (CD25^+/−^IL-17^−/−^IFN-γ^−/−^). Similar to parental strains, no gender predilection was seen (not shown).

**Figure 6:**
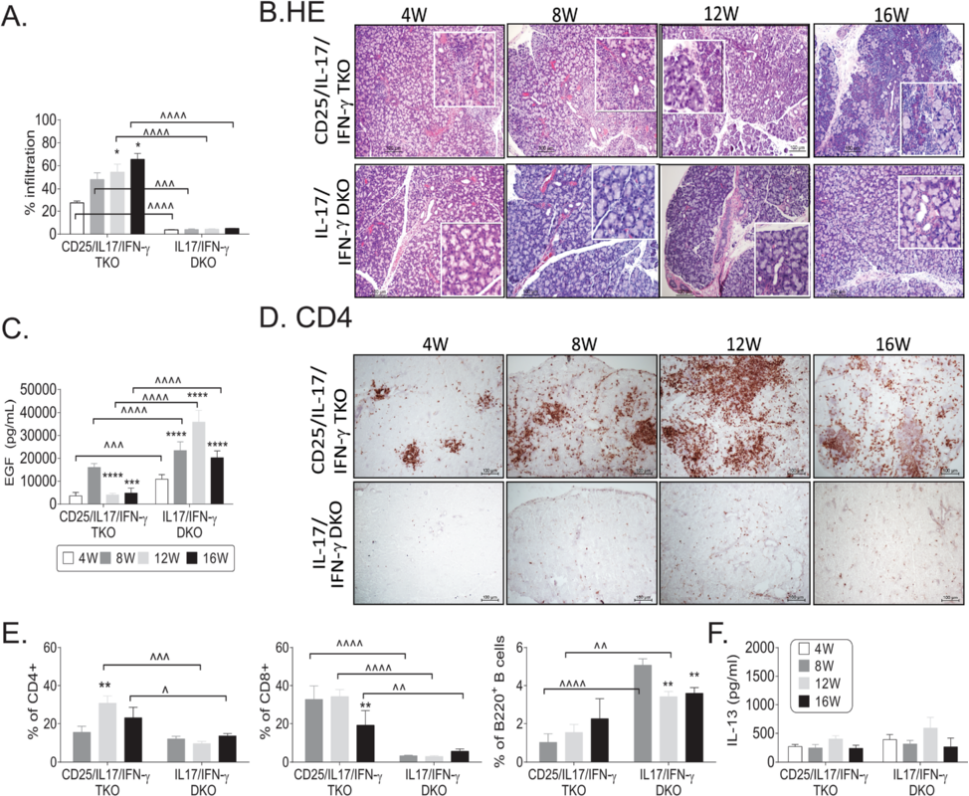
**Less severe SS-like disease in CD25/IL-17/IFN-** **γ** **TKO mice. (A)** Percentage of lymphocytic infiltration measured in digital images of H&E-stained LG sections from CD25/IL-17/IFN-γ TKO and IL-17/IFN-γ DKO mice aged 4 to 16 weeks (W). **(B)** Representative images of H&E-stained LG sections from CD25/IL-17/IFN-γ TKO and IL-17/IFN-γ DKO mice aged 4 to 16 weeks (W). The square is a higher magnification of the area underneath. Scale bar = 100 μm. **(C)** EGF concentration measured in tear washings from different strains (n = 6 to 20 per age/strain). **(D)** Representative images of immunohistochemistry for LG sections stained for CD4^+^ T cells (red) CD25/IL-17/IFN-γ TKO and IL-17/IFN-γ DKO mice aged 4 to 16 W. Scale bar = 100 μm. **(E)** Percentage of CD4^+^ T, CD8^+^ T and B220^+^ cells gated in flow cytometry from LG isolated from CD25/IL-17/IFN-γ TKO and IL-17/IFN-γ DKO LG isolates from mice aged 8 to 16 W. Data are presented as mean ± SEM of six individual samples/strain/age. **(F)** Concentration of IL-13 in serum from CD25/IL-17/IFN-γ TKO and IL-17/IFN-γ DKO mice aged 4 to 16 W. Data are presented as mean ± SEM of five to ten individual samples/strains/age. ^*^ *P* <0.05; ^**^ *P* <0.01, ^***^ *P* <0.001; ^****^ *P* <0.0001 within strain comparison vs. 4 W **(B and C)** or 8 W **(E and F)**. ^^^ *P* <0.05; ^^^^ *P* <0.01, ^^^^^ *P* <0.001; ^^^^^^ *P* <0.0001 interstrain comparison. CD25/IL-17/IFN-γ TKO, CD25^−/−^IL-17^−/−^IFN-γ−/−; DKO, double knockout; EGF, epidermal growth factor; Fas-L, Fas-ligand; H&E, hematoxylin and eosin; IFN-γ, interferon-gamma; IL-17/IFN-γ DKO, CD25^+/−^IL-17^−/−^IFN-γ^−/−^; IL, interleukin; LG, lacrimal gland; SEM, standard error of the mean; SS, Sjögren’s syndrome; TKO, triple knockout.

IL-17/IFN-γ DKO had normal-appearing LG with minimal lymphocytic infiltration at all ages (4 to 16 W), while in CD25/IL-17/IFN-γ TKO mice areas of immune infiltrates were seen at 8 W (20% of total LG) and progressively increased up to 16 W, compromising about 60% of the LG area (Figure 6A and B). Tear EGF concentration in CD25/IL-17/IFN-γ TKO and IL-17/IFN-γ DKO mice was in the normal range at 8 W; however, a significant decline in tear EGF concentration was seen in CD25/IL-17/IFN-γ TKO as they aged to 16 W (Figure 6C).

Histologically, a mix of CD4^+^, CD8^+^ and CD19^+^ cells was seen in the LG, with a greater number of CD4^+^ (Figure 6D) and CD8^+^ (not shown) in the CD25/IL-17/IFN-γ TKO compared to IL-17/IFN-γ DKO mice. Flow cytometric analyses indicated that the immune cell aggregates consisted of CD4^+^, CD8^+^ T cells, and B lymphocytes (Figure 6E). There was an increased percentage of CD4^+^ and CD8^+^ in CD25/IL-17/IFN-γ TKO compared to IL-17/IFN-γ DKO mice (8 W to 16 W), with a predominance of CD8^+^ cells. There was a significant increase in CD4^+^ cells at 12 W compared to 8 W in CD25/IL-17/IFN-γ TKO mice, paralleling increased lymphocytic infiltration seen in histologic sections. IL-17/IFN-γ DKO had the highest percentage of B cells (Figure 6E).

Gene expression could not be evaluated in the LG of CD25/IL-17/IFN-γ TKO mice due to difficultly breeding of this strain. To investigate whether the CD25/IL-17/IFN-γ TKO mice had a compensatory elevation of IL-13 as seen in CD25KO mice, we measured serum IL-13 concentration. Our results demonstrated no difference in IL-13 levels with age or between CD25/IL-17/IFN-γ TKO and IL-17/IFN-γ DKO groups (Figure 6F).

Because we observed delayed and less severe dacryoadenitis when IFN-γ was deleted in the CD25KO mice, we sought to investigate whether IFN-γR expression and TUNEL^+^ cells were also decreased in the CD25/IL-17/IFN-γ TKO mice. IFN-γR immunostaining in LG at 4 and 8 W showed very minimal immunoreactivity in glandular tissue in IL-17/IFNγ DKO (Figure 5A) while a mild expression was seen in CD25/IL-17/IFN-γ TKO mice.

IL-17/IFN-γ DKO had similar levels of TUNEL^+^ cells to nonimmune C57BL/6 mice (53.25 ± 16.04 vs. 32 ± 6.75 TUNEL^+^ cells/20X field, respectively, *P* >0.05, Figure 5E and F), while CD25/IL-17/IFN-γ TKO mice had mild dispersion of TUNEL^+^ cells in the LG at 8 W, although it was still much lower than CD25KO and CD25/IL-17 DKO mice (215.67 ± 23.24 vs. 429 ± 64.59 and 1,100 ± 101.85 TUNEL^+^ cells/20X field, *P* <0.001 and *P* <0.0001, respectively, Figure 5E and F).

These results confirm that increased IFN-γR expression in CD25/IL-17 DKO is associated with the increased apoptosis and increased severity of dacryoadenitis in the CD25KO mouse model of SS. Even though CD25/IL-17/IFN-γ TKO mice had increased disease compared to IL-17/IFN-γ DKO, it was much less severe than the CD25KO and CD25/IL-17 DKO parental strains, demonstrating that IFN-γ has a greater pathogenic role than IL-17A.

## Discussion

Our results showed that deletion of IL-17A in the CD25KO model accelerated onset and severity of dacryoadenitis. This was accompanied by increased CD4^+^ and CD8^+^ T cell infiltration and increased expression of inflammatory cytokines, IFN-γR, Fas-L and TRAIL, lower levels of local and systemic IL-13 and increased LG apoptosis than CD25KO mice. CD25/IFN-γ DKO mice had lower activity levels of caspase-3, and -9 and significant lower number of TUNEL^+^ cells. Deletion of IFN-γ from CD25/IL-17 DKO (generating a CD25/IL-17/IFN-γ TKO) had an inverse effect to CD25/IL-17 DKO, preserving glandular tissue and delaying dacryoadenitis severity.

Tear dysfunction (reduced tear production or increased inflammatory tear content) is responsible for some severe forms of dry eye and may be caused in part by cytokines such as IL-1 that are released by infiltrating cells or stressed glandular epithelial cells. In the present study, IL-1β transcripts in CD25/IL-17 DKO mice at all ages were significantly higher than in CD25KO mice and paralleled the lymphocytic infiltration in these mice. The proinflammatory and apoptotic effects of IL-1 in the LG have been extensively studied [30,31]. A single injection of IL-1 into the extraorbital LG induced a mild decrease in LG secretion, while inducing a robust, yet reversible (7 to 13 days) inflammatory response that led to destruction of LG acinar epithelial cells [30]. Similar to these studies, the increased IL-1β in LG of CD25/IL-17 DKO may be critical in inducing inflammatory response in these CD25/IL-17 DKO mice.

Interestingly, both IL-1 and TNF-α can upregulate expression of IFN-γR [32] and IL-1 was significantly increased in the CD25/IL-17 DKO mice. Increased expression of IFN-γR in CD25/IL-17 DKO may sensitize to the effects of IFN-γ that is secreted by T cells and NK cells. Deletion of IFN-γR in the autoimmune MRL/lpr mice prevented apoptosis of the tubular epithelial cells and development and severity of kidney autoimmune disease [33]. Treatment of colonic cell lines with IFN-γ increased the sensitivity of colonic epithelial cells to diverse apoptotic stimuli in concert, via upregulation of caspase-1 [34].

MHC II expression can be induced by IFN-γ. LG epithelial cells trigger or exacerbate lacrimal autoimmune disease by presentation of autoantigens via MHC II [35]. In our study, higher MHC II transcripts were found in both CD25KO and CD25/IL-17 DKO mice suggesting a greater potential of MHC II^+^ cells to initiate autoimmunity.

The CD25KO model is a unique system where autoimmunity develops spontaneously in many organs, including salivary gland [6], lacrimal gland [7] and liver [11] and it is age-dependent [36]. IL-17A serum levels [11] and transcripts in LG [7] of CD25KO mice peaked early (around 8 to 12 W) suggesting that IL-17A could be participating in early disease development. The number of autoimmune diseases that have been shown to have an IL-17 component has increased after discovery of IL-17, including SS [37]. Elevated levels of IL-17A protein were found in minor salivary glands and conjunctiva of SS patients compared to normal control subjects [21,38]. Our group and others showed that neutralization of IL-17A ameliorates corneal barrier disruption in response to desiccating stress [21,39] and decreases expression of matrix metalloproteinase (MMP)-3 and MMP-9 mRNA in the corneal epithelium [21]. Despite the fact that increased IL-17 mRNA and protein has been found in LG of mouse models of SS [7,37,40], the exact role of IL-17 in the LG remains unsolved. Most of the published studies describing a pathogenic role for IL-17A evaluated the salivary gland without description of the LG. Adenovirus-mediated transfer of an IL-17A gene vector to the salivary gland ducts of nonimmune C57BL/6 mice was sufficient to induce an SS-like disease, inclusive of lymphocytic infiltration and decreased salivary flow [41]. Adenovirus-mediated transfer of IL-17R:FC decreased sialodenitis in the susceptible C57BL/6.NOD-Aec1Aec2 strain [42]. Furthermore, IL-17KO mice that adoptively received Th17 cells from mice immunized with salivary glands extracts had reduced saliva secretion, elevated autoantibody production and pronounced inflammation and tissue damage in the submandibular gland [43]. However, in most of these inducible models, IL-17 response was solely induced without the confounding interaction with other T cell cytokines, such as IFN-γ and IL-13.

The role of IL-17A may also be tissue specific; in dextran sodium sulphate induced-colitis, IL-17 is protective, since neutralization of IL-17 worsened colitis [44] while CD25/IL-17 DKO mice had more severe autoimmune biliary disease [45]. Regarding the LG, genetic deletion of IL-17A in an elevated unchecked IFN-γ environment in the CD25KO mice did not abolish development of autoimmunity; rather, it accelerated its onset as evidenced by increased LG lymphocytic infiltration and reduced function measured by decreased EGF concentration in tears.

In some experimental models, such as experimental autoimmune uveitis (EAU) both IL-17 and IFN-γ seem to be pathologic [46-48]. Our results evaluating the CD25/IL-17/IFN-γ TKO showed that IFN-γ is more pathogenic than IL-17A, since deletion of IFN-γ in the CD25/IL-17 DKO reversed the acute and severe changes observed in this model, also decreasing IFN-γR expression and decreasing apoptotic cells. However, because these mice still have a defective IL-2 signaling, disease was not completely abolished. IL-2 is a master regulator of the immune system and it is involved in generation of Tregs, activation-induced cell death and suppression of Th17 generation [6,10,49] and it is critical for Th1 function. Recent studies showed that reduced Treg cell function may contribute to the development of multiorgan systemic autoimmune disease [6]. Spontaneous development of dacryoadenitis and keratoconjunctivitis is observed in mouse strains with defective Treg functions [50,51]. In NOD mice, the deletion of Treg cells worsens autoimmune activity [52]. In an attempt to dissect the individual contributions of lack of Tregs and increased life span of activated T cells, crossbreeding of CD25KO with B6.lpr mice improved survival and decreased colonic and LG infiltration, demonstrating that some tissue effects are independent of Tregs [10]. We observed increased Fas-L and TRAIL expression in CD25/IL-17 DKO mice, which have the greatest apoptosis, glandular tissue loss and lymphocytic infiltration compared to parental strains. This is extremely important as increased Fas-L expression in T cells damages adjacent cells, as demonstrated in CD25/B6.lpr mice and other systems [25-27].

We observed higher EGF concentration levels in IL-17/IFN-γ DKO than IL-17KO. It is possible that the LG acini may be very sensitive to low amounts of IFN-γ and may account for the differences observed in these two strains. IFN-γ has been described to be critical for LG and salivary gland development [53,54]. IFN-γ induces apoptosis, death and structural changes to human salivary gland cultures [55,56]. In an *in vitro* model, mouse conjunctival explant cultures are exquisitely sensitive to IFN-γ: minute concentrations of IFN-γ at the early days of culture are sufficient to change morphology and induce apoptosis [57]. Our findings describing the CD25/IL-17/IFN-γ TKO demonstrate that IFN-γ appears to make a greater contribution to LG inflammation and secretory dysfunction than IL-17, which make actually have some protective functions. We believe that disrupting the balance of T helper cytokines can alter disease manifestations.

The cross-regulation of IL-13 and IFN-γ has been well established in *in vitro* and *in vivo* models of asthma [14,58,59] and IL-13 has been shown to be protective to epithelium *in vitro* [18,19,23]. IL-13 can be produced by Th2 cells and also NK cells [60,61]. We have also shown that IL-13 is critical for maintenance of conjunctival goblet cell. IL-13KO have lower goblet cell density at baseline and exogenous administration of IL-13 to wild-type mice is capable of rescuing goblet cell after desiccation-induced stress [60]. The conjunctiva, similarly to the airway epithelia, is very susceptible to apoptotic effects of IFN-γ; IFN-γKO are resistant to desiccation-induced goblet cell loss while exogenous subconjunctival injection of IFN-γ in these mice recapitulate the phenotype observed in wild-type mice [62]. In the parental CD25KO strain, there is a spontaneous proliferation of Th1, Th17 and Th2 cells. IL-13 has been shown to be antiapoptotic on colonic and airway epithelial as well as conjunctival fibroblasts [18,19,23]. Our results in this manuscript suggest that in a low IL-13 environment in the presence of increased IFN-γ and IFN-γR contribute to increased glandular apoptosis.

Our results showed lower activity levels of caspases-3 and -9 and lower number of TUNEL^+^ cells in the CD25KO mice compared to the CD25/IL-17DKO and even lower levels in CD25/IFN-γ DKO compared to CD25KO mice, demonstrating that IFN-γ is critical for the glandular apoptosis. CD25/IFN-γ DKO mice had delayed glandular destruction and preserved secretory function at 8 W [13], in agreement with findings in the NOD model of SS where deletion of IFN-γ improved sialodenitis, decreased caspase-3 activity and TUNEL^+^ cells compared to parental NOD strain [12]. We have also shown that in our inducible dry eye model neutralization of IFN-γ decreased caspase-3 and caspase-8 and increased conjunctival goblet density [24,29]. Desiccating stress by itself did not increase caspase-9; however, exogenous administration of IFN-γ during desiccating stress upregulated caspase-9 RNA [24,29]. These results indicate that IFN-γ actively participates in the dacryoadenitis in the CD25KO model through inducing the intrinsic (caspase-9) and common apoptotic (caspase-3) pathways.

We wish to emphasize that this is the first report of using a combination several DKOs and one TKO in an attempt to dissect individual contributions of IL-17A and IFN-γ in dacryoadenitis in a murine model. There are certainly some limitations to our study. The disease and LG disarrangement is rather severe and accelerated in the CD25KO mouse, while in humans it may be more insidious and chronic in nature. Although we could not directly prove the involvement of IL-13 in protecting lacrimal epithelium we speculate that an intact Th2 and B cell response in CD25KO mice delays development of pathological infiltrates and destruction of parenchymal tissue based on our findings. Additional studies will certainly help dissect the exact contribution of IL-13 in the lacrimal gland immunopathology that develops in the CD25KO mice.

## Conclusions

**Figure 7:**
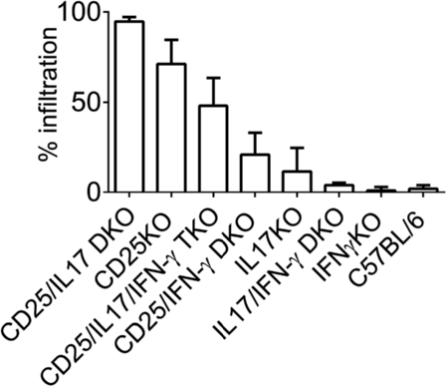
**Severity of dacryoadenitis among autoimmune and nonimmune strains at 8 weeks of age.** Total infiltration was measured in paraffin-embedded H&E-stained LG sections as described in materials and methods. H&E, hematoxylin and eosin; LG, lacrimal gland.

Taking in consideration what we learned through selective removing genes encoding pathogenic factors in the CD25KO strain, we assembled the graph in Figure 7 that grades severity based on the percentage of total lymphocytic infiltration of the LG at 8 W. A gradient of LG infiltration can be observed, where the CD25/IL-17 DKO has the worst disease, while the CD25/IFN-γ DKO [13] is on the other end of the spectrum with much less severe disease. Severity of LG infiltration in the IL-17/IFN-γ DKO and IFN-γKO is similar to that seen in C57BL/6 mice. These results suggest that therapies targeting both cytokines may be more beneficial in the treatment of SS than targeting a single cytokine.

## Abbreviations

^+^: Positive
Aec1Aec2: autoimmune exocrinopathy NOD mice in C57BL/6 background
BSA: bovine serum albumin
CD25/IFN-γ DKO: CD25^−/−^IFN-γ^−/−^
CD25/IL-17/IFN-γ TKO: CD25^−/−^IL-17^−/−^IFN-γ−/−
CD25KO: CD25 knockout
DKO: double knockout
EAU: experimental autoimmune uveitis
EGF: epidermal growth factor
ELISA: enzyme-linked immunosorbent assay
Fas-L: Fas-ligand
H&E: hematoxylin and eosin
HPRT-1: hypoxanthine phosphoribosyltransferase 1
IFN-γ: interferon-gamma
IHC: immunohistochemistry
IgG: immunoglobulin G
IL-17/IFN-γ DKO: CD25^+/−^IL-17^−/−^IFN-γ^−/−^
IL: interleukin
KO: knockout
LG: lacrimal gland
MHC-II: major histocompatibility complex class II
MMP: matrix metalloproteinase
NK: natural killer cells
NOD: nonobese diabetic
PBS: phosphate-buffered saline
R: receptor
SS: Sjögren’s syndrome
Th: T helper
TKO: triple knockout
TNF: tumor necrosis factor
TRAIL: TNF-related apoptosis-inducing ligand
Treg: T regulatory cells
TUNEL: terminal deoxynucleotidyl transferase dUTP nick end labeling
W: week
